# Investigating the Suitability of Carbon Nanotube Reinforced Polymer in Transcatheter Valve Applications

**DOI:** 10.1007/s13239-017-0313-2

**Published:** 2017-06-16

**Authors:** Monica M. Rozeik, David J. Wheatley, Terence Gourlay

**Affiliations:** Department of Biomedical Engineering, Wolfson Centre, University of Strathclyde, 106 Rottenrow, Glasgow, G4 0NW UK

**Keywords:** Composite, Leaflet material, Delivery profile

## Abstract

The current delivery size of transcatheter aortic valves, limited by the thickness of their pericardial leaflets, correlates with a high prevalence of major vascular complications. Polyurethane valves can be developed to a fraction of the thickness of pericardial valves through the addition of carbon nanotubes to reinforce their leaflets. This study investigates the suitability of a novel carbon nanotube reinforced leaflet to reduce the delivery profile of transcatheter aortic valves. Carbon nanotube polyurethane composites were developed with thicknesses of 50 *μ*m and their mechanical properties were determined in relation to various environmental effects. The composites demonstrated improvements to the material stiffness, particularly at increasing strain rates compared to the neat polymer. However, increasing nanotube concentrations significantly decreased the fatigue life of the composites. Key findings highlighted a potential for carbon nanotube reinforcement in valve replacement which experience very high strain rates during the cardiac cycle. Further testing is needed to achieve a strong nanotube–matrix interface which will prolong the cyclic fatigue life and further strengthen tensile properties. Testing on the durability and haemocompatibility of these composite heart valves are ongoing.

## Introduction

Since the first human implantation in 2002, transcatheter aortic valve implantation (TAVI) has gained increasing popularity, providing an alternative solution to high risk patients suffering severe aortic stenosis but who cannot undergo conventional open heart surgery for valve replacement.^4^, ^8^, ^26^, ^32^ Between the first and third generation, the size of delivery catheters for a retrograde transfemoral (TF) approach has substantially reduced from 25 to 16 Fr.^4^, ^5^, ^13^–^15^ Despite this, major vascular bleeding and trauma associated with the size of the delivery catheter are the most frequently reported complications with TF-TAVI.^17^, ^29^

The overall size of the delivery system can be decreased by reducing the thickness of the valve leaflets.^18^ Polymers are a promising alternative to pericardium and can be used to manufacture leaflets with a great deal of flexibility in design and with controlled precision to produce ultra-low (<100 *µ*m) leaflet thicknesses.^10^, ^16^ Unquestionably a reduction in thickness risks compromising the mechanical integrity of the material. It is well recognized that native leaflets are reinforced with elastin and collagen fibers to facilitate leaflet flexion and coaptation. Despite this, very few studies on polymeric valves have also considered fiber reinforcement.^2^, ^6^, ^9^ Synthetic fibers like polypropylene have low length to diameter aspect ratios which can result in a weak fiber/matrix interface. They also reduce leaflet flexibility and limit the manufacturing techniques possible such as injection molding where the fibers would be too large for the extruder or dip coating where fibers can migrate.

In this regard, carbon nanotubes can make excellent potential composite fillers as their nano-diameters and micrometer lengths give them very high aspect ratios whilst retaining their flexibility which is critical to handling the dynamic demands of the heart.^3^, ^30^ Moreover, the haemocompatibility benefits of polyurethane-nanotube composites have been promising; promoting cell growth and reducing platelet attachment and changes to platelet morphology compared to the neat polymer.^23^

This study will investigate the suitability of carbon nanotube composites as leaflet materials for synthetic heart valves by examining the influence of various environmental conditions including strain rates and hydration on their mechanical properties. The findings of this study will be used to determine the best approach to take moving forward in the development of polymer-nanotube composite leaflets for TAVI.

## Materials and Methods

### Carbon Nanotubes

Multi-walled carbon nanotubes with a purity of >90% and having low and high aspect ratios were obtained from Sigma-Aldrich (Dorset, UK). The low aspect ratio nanotubes had diameter × length dimensions of 110–170 nm × 5–9 *μ*m and the high aspect ratio nanotubes had dimensions of 10–15 nm × 0.1–10 *μ*m. The chemical composition of the two nanotube types was determined using a WITec Alpha 300R confocal Raman microscope for all spectral acquisitions at an excitation wavelength of 532 nm. Spectra were collected using an Olympus MPLanFL N 100×/NA 0.9 objective lens and the data was analysed using WITec Project software, version 2.02. Scanning electron microscopy (SEM) images of the nanotubes in their raw powder form mounted on conductive carbon films were taken using a FEI Sirion 200 field-emission gun scanning electron microscope (FEGSEM).

### Synthesis of Nanotube Reinforced Polyurethane Composites

A 15% w/v polymeric solution was made by dissolving pellets of polycarbonate urethane (Carbothane^®^ PC3595A, Lubrizol, Wickliffe, Ohio) in dimethylacetamide solvent. Nanotubes were then added to the solution to make composite concentrations of 0.125, 0.25, 0.5 and 1.0% w/w. The nanotubes were dispersed in the polymeric solution using an ultrasonic bath at 37 kHz at 50 °C to decrease the viscosity. The ideal dispersion rate was determined from the tensile properties of 0.5 and 1.0% nanotube composites after 1, 2, 2.5, 5 and 7 h of dispersion. Composite films were developed having thicknesses of approximately 50 *μ*m and cut into 3.5 × 30 mm strips. Images of the surface topography of 0.5 and 1.0% composites were acquired using an Asylum Research MFP-3D atomic force microscope (AFM) in contact mode using tetrahedral cantilever tips (OMCL AC160TS). Optical microscopy images were taken with a Carl Zeiss Axio Scope microscope at 60× magnification to show the relative dispersion of nanotubes.

### Mechanical Property Testing

Tensile testing was conducted using the Bose ElectroForce 3200 universal testing machine (Bose Corporation, Framingham, USA), with a gauge length of 11 mm and a displacement rate of 1 mm/s. The strips were stretched within their elastic limits to 5 mm (45% strain) and held for 30 s to record the stress relaxation. The 5% secant modulus was determined from the straight line of the stress–strain graph from 0 to 5% strain. The room temperature was maintained at 23 ± 1 °C.

The environmental effects on the composite mechanical properties determined were:

1. *Effect of hydration* 0 and 1.0% composite strips were immersed in a container of filtered distilled water for 9 months and tested intermittently.
2. *Effect of tensile strain rates* 0–1.0% composite strips were tested at displacement strain rates of 1, 2, 5 and 10 mm/s to 45% strain by loading the sample and returning the crosshead to its default position before loading the next strain rate.
3. *Effect of cyclic fatigue* Composite strips were cycled between 0 and 70% at a frequency of 20 Hz until failure (defined as the number of cycles to complete rupture).
4. *Effect of sterilization* 0 and 1.0% composite strips were subjected to a gamma radiation dose of 32.5 kGy and their tensile properties were determined at 2, 14, 42 and 93 days post sterilization.

### Statistical Analysis

All results are reported as mean (± standard error of the mean, SE). Data were analysed using a one-way ANOVA test and statistical significance was defined as *p* < 0.05.

## Results

### Characterization of Nanotubes

**Figure 1:**
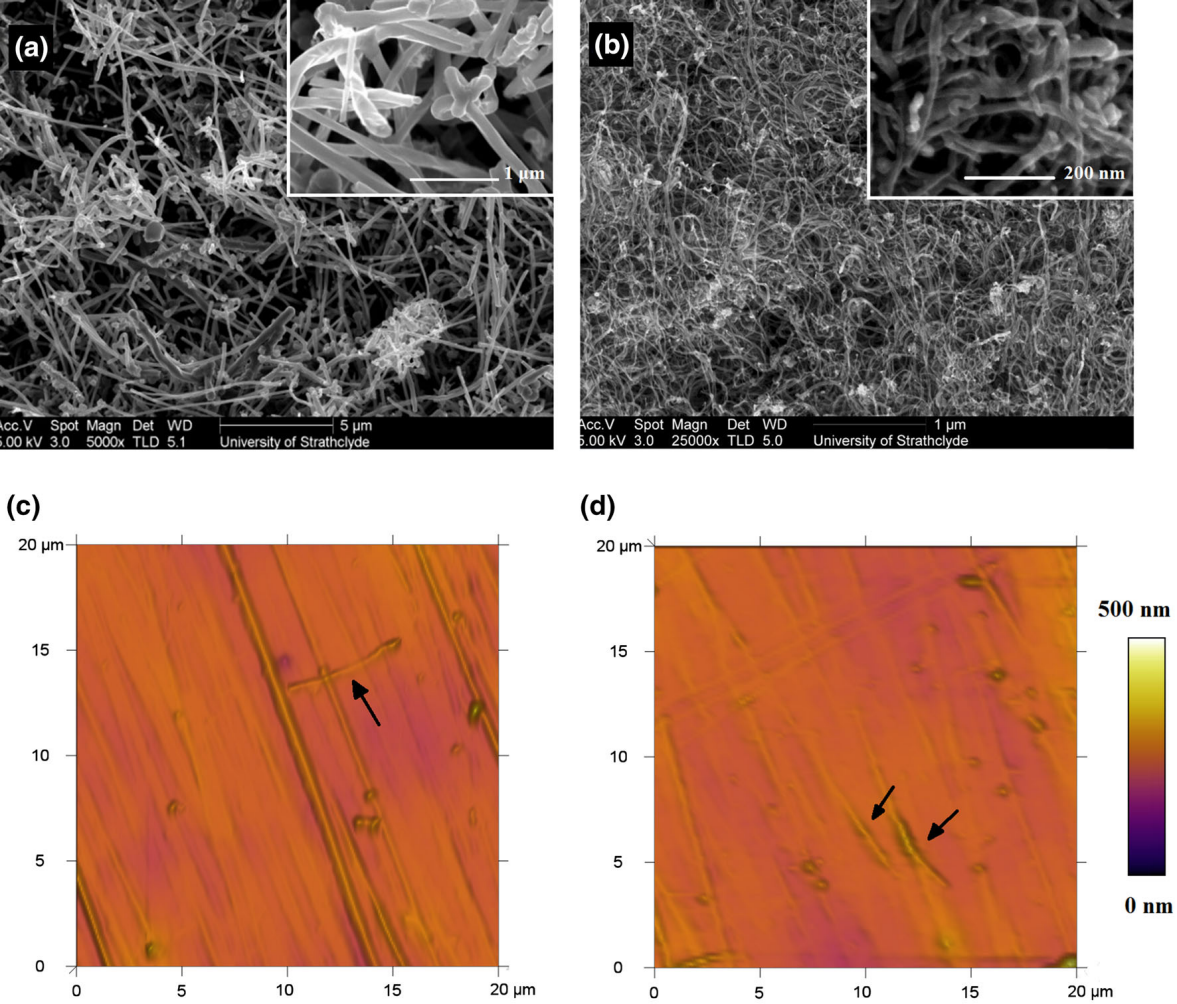
Microscopy images of the nanotubes; typical SEM images of the (a) low (5000×) and (b) high aspect ratio (25,000×) nanotubes in their raw powder forms, and respective images close up at 20,000× and 100,000× magnification. Typical AFM topography images of (c) 0.5% and (d) 1.0% w/w low aspect nanotubes in PC3595A polyurethane. Black arrows highlight nanotubes imbedded within the polymer matrix.

Scanning electron microscope (SEM) images of low and high aspect ratio nanotubes in their raw powder form are shown in Fig. 1. The high aspect ratio nanotubes were considerably smaller and intermeshed which suggested that they had a higher tendency to agglomerate.

**Figure 2:**
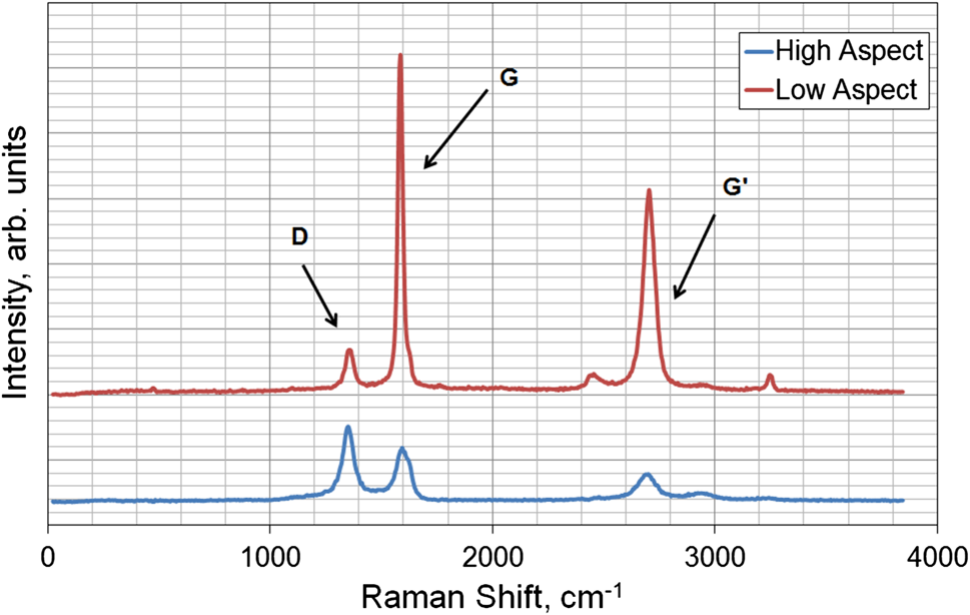
Typical Raman spectra of low and high aspect ratio nanotubes showing the positions of the *D*, *G* and *G*′ bands.

**Figure 3:**
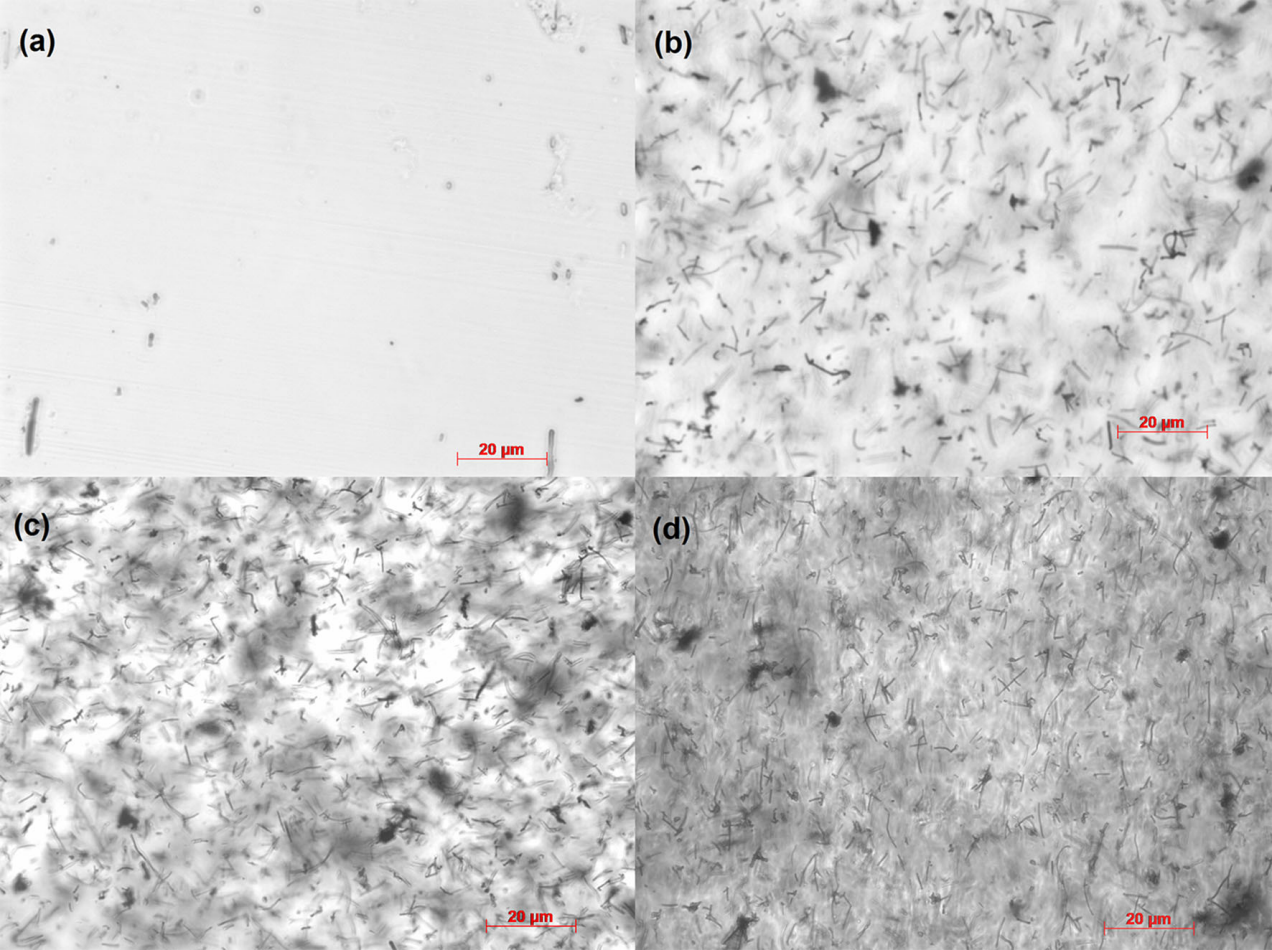
Representative bright-field microscopy images of the distribution of nanotubes within the polymer matrix in (a) 0%, (b) 0.125%, (c) 0.5%, and (d) 1.0% w/w composites. Scale bars represent 20 *µ*m.

The ratio of the D band (disorder in carbon bonds) to the G band (stretching mode of carbon-carbon bonds in the graphene plane) at 1350 cm^−1^ and 1580 cm^−1^ respectively can be used to determine the relative purity of the carbon nanotubes, with a lower D band intensity indicating a higher purity. Another peak at 2700 cm^−1^ (*G*′ band) is also observed, caused by interactions between the nanotube layers. This is noticeably higher in the low aspect ratio nanotubes as they have larger diameters and consequently more tube layers. The spectra for the atomic structure of the two different aspect ratio nanotubes is shown in Fig. 2. The mean (SE) peak intensity band area ratio D/G was lower in the low aspect ratio nanotubes than for the high aspect ratio nanotubes, having a ratio of 0.11 (0.01) compared to 1.42 (0.08). This suggested that despite the higher reinforcement potential of the high aspect ratio nanotubes, they had greater defects than low-aspect ones. Accordingly, all composite testing was conducted using low aspect ratio nanotubes due to their higher purity and less tendency to agglomerate. Bright-field microscopy images at 60x magnification of the composites are shown in Fig. 3. Some nanotube bundles can be observed in the higher composite concentrations but overall show an even distribution of the fibers across the polymer matrix.

### Mechanical Testing

**Figure 4:**
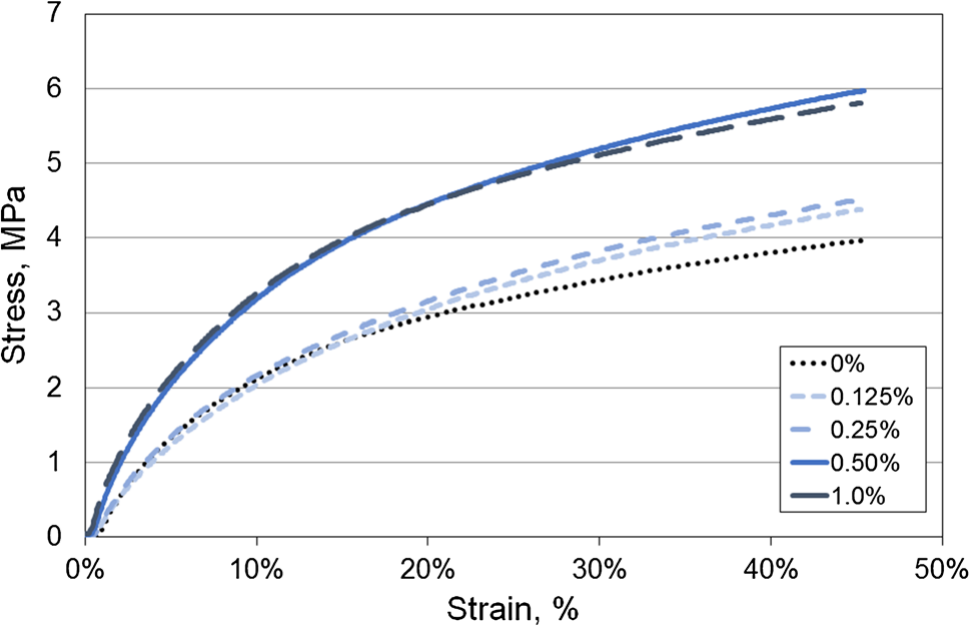
Typical stress–strain curves exhibited in Carbothane^®^ PC3595A with increasing nanotube concentration from 0.125 to 1.0% w/w.

The mean (SE) secant modulus of the composites with varying concentrations from 0%-1.0% w/w of nanotubes are shown in Table 1. The modulus of the neat polymer increased significantly (*p* < 0.05) from 36.8 (1.2) to 41.1 (1.3) and 42.0 (0.8) MPa when 0.5 and 1.0% w/w nanotubes were added but no significant change was noted between the 0.5 and 1.0% composites. Concentrations below 0.5% w/w appeared to have a detrimental effect on the composite secant modulus. Typical stress–strain response of the composites are shown in Fig. 4. The mechanical properties of the polymer improved with increasing nanotube concentration at higher strains.

**Table 1:** Summary of results (mean ± SE) for the tensile tests and cycles to failure at 70% strain.

Concentration (%)	*N*	S-Mod (MPa)	SR (%)	Thickness (*µ*m)	Cycles to failure (×10^6^)	
					Mean	Range
0.000	16	36.8 ± 1.2	43.4 ± 0.6	50.1 ± 1.2	1.04 ± 0.12	0.36–1.70
0.125	6	24.4 ± 0.9	41.5 ± 0.1	39.4 ± 2.3	0.99 ± 0.19	0.48–1.61
0.250	6	28.4 ± 0.9	42.1 ± 0.1	41.1 ± 1.3	0.69 ± 0.10	0.48–0.94
0.500	16	41.1 ± 1.3	44.2 ± 0.7	49.1 ± 1.7	0.44 ± 0.16	0.13–1.23
1.000	16	42.0 ± 0.8	41.9 ± 0.6	49.6 ± 1.4	0.30 ± 0.05	0.03–0.59

S-Mod—5% secant modulus, SR—stress relaxation

### Effect of Dispersion Time

**Figure 5:**
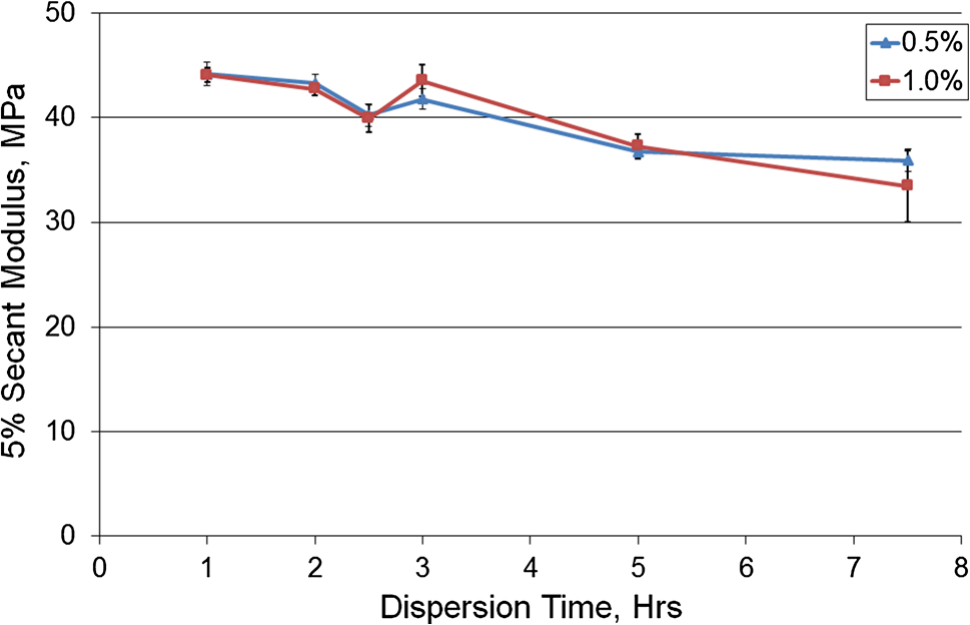
Mean secant modulus against the dispersion time (hours) in an ultrasonic bath for 0.5 and 1.0% composites (*n* = 5). Error bars represent ± SE.

The mean secant modulus of the 0.5 and 1.0% w/w composites after dispersing in an ultrasonic bath for 1–7.5 h are shown in Fig. 5. Increasing the dispersion time decreased the secant modulus.

### Effect of Immersion in Water

**Figure 6:**
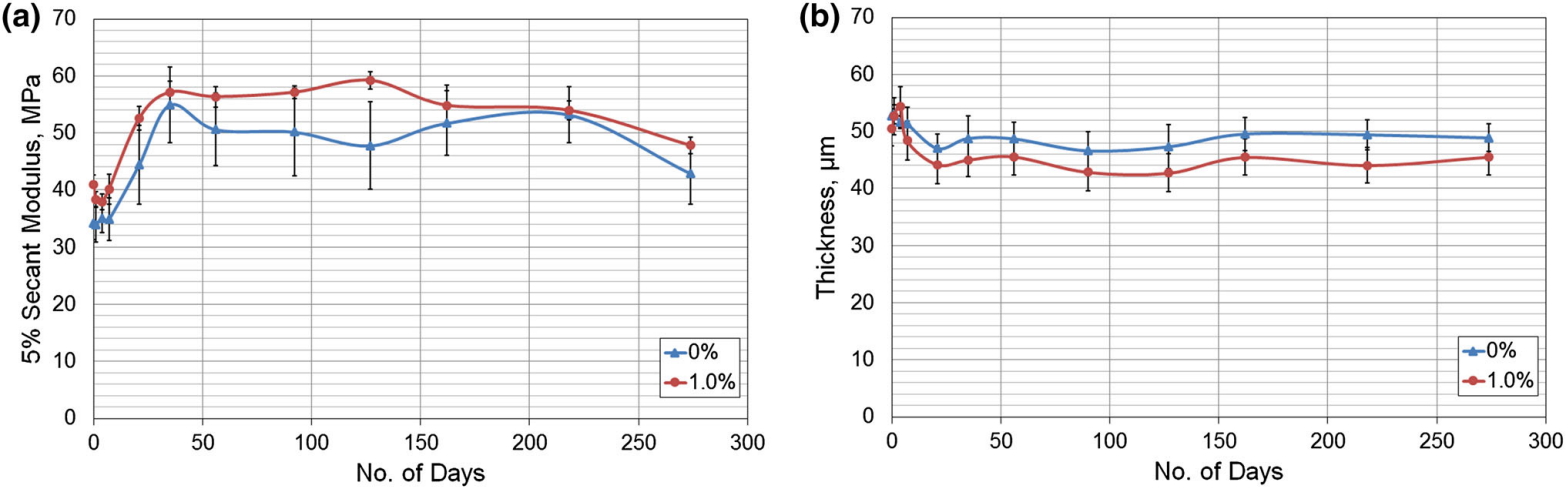
(a) Secant Modulus at 5% strain of 0 and 1.0% composites after immersion in distilled water for several days (*n* = 5). (b) Sample thickness (*µ*m) with increasing time immersed in water (*n* = 6). Error bars represent ± SE.

The mean (SE) secant modulus of the 0 and 1.0% composites is shown in Fig. 6a. A significant (*p* < 0.05) increase in modulus was observed between days 7 and 35 in the neat polymer and between days 7 and 21 in the composite, which suggested that the composites reached saturation sooner than the neat polymer. There was also a larger variation in the modulus recorded for the neat polymer compared to the composite (average error of the mean was 5.3 MPa compared to 2.1 MPa respectively). Despite the slight decrease in thickness over the first 7 days, there were no significant changes in thickness over time, suggesting that swelling did not occur (Fig. 6b). The initial thickness of the neat polymer was 52.7 (1.2) *μ*m compared to a thickness of 50.5 (3.1) *μ*m, but became on average 5.3 *μ*m thicker than the composite at approximately 30 weeks, although this was not significant.

### Effect of Mechanical Properties on Strain Rate

**Figure 7:**
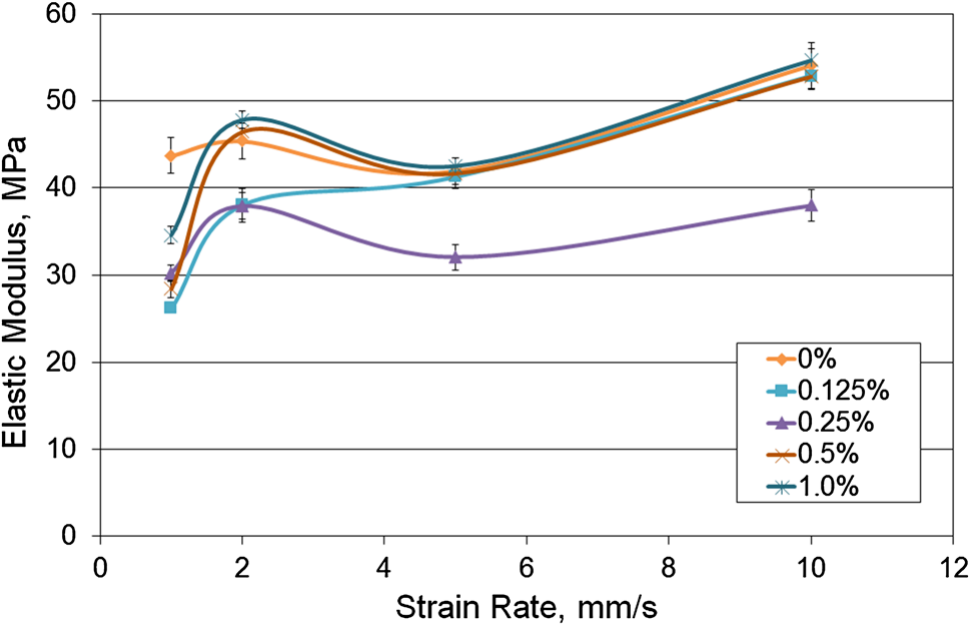
Elastic modulus between 0.5 and 1.0 MPa stress with increasing strain rate and nanotube content (*n* > 4).

The stiffness of the composites was determined from the linear region of the graph between 0.5 and 1.0 MPa for varying nanotube contents at 1, 2, 5 and 10 mm/s strain rate (Fig. 7). An initial attempt was made to derive the secant modulus but this produced an inaccuracy due to the slack in the samples following each loading step. The neat polymer of this testing batch had a higher elastic modulus than the composites, suggesting large variation can occur between composite batches. Despite this, with increasing strain rates, the mechanical properties of the composites increased significantly (*p* < 0.05). The elastic modulus of the 1.0% composite was higher than the neat at a tensile rate of 10 mm/s.

### Effect on Cyclic Fatigue

Data pooled from the tensile tests, sonication and sterilization studies on the secant modulus, stress relaxation and thickness is shown in Table 1. Some samples failed prematurely and were not included in the fatigue data. With the exception of the 0.125% composite which had similar fatigue properties to the neat polymer, a decrease in fatigue life with increasing nanotube concentration was observed. Scanning electron microscope images of the ruptured specimens showed surface cracking of the neat polymer which was not evident in the 0.125 and 0.25% composites. The thickness of the strips ranged from 39.4 to 50.1 *μ*m but did not appear to influence fatigue life.

### Effect of Gamma Sterilization

**Figure 8:**
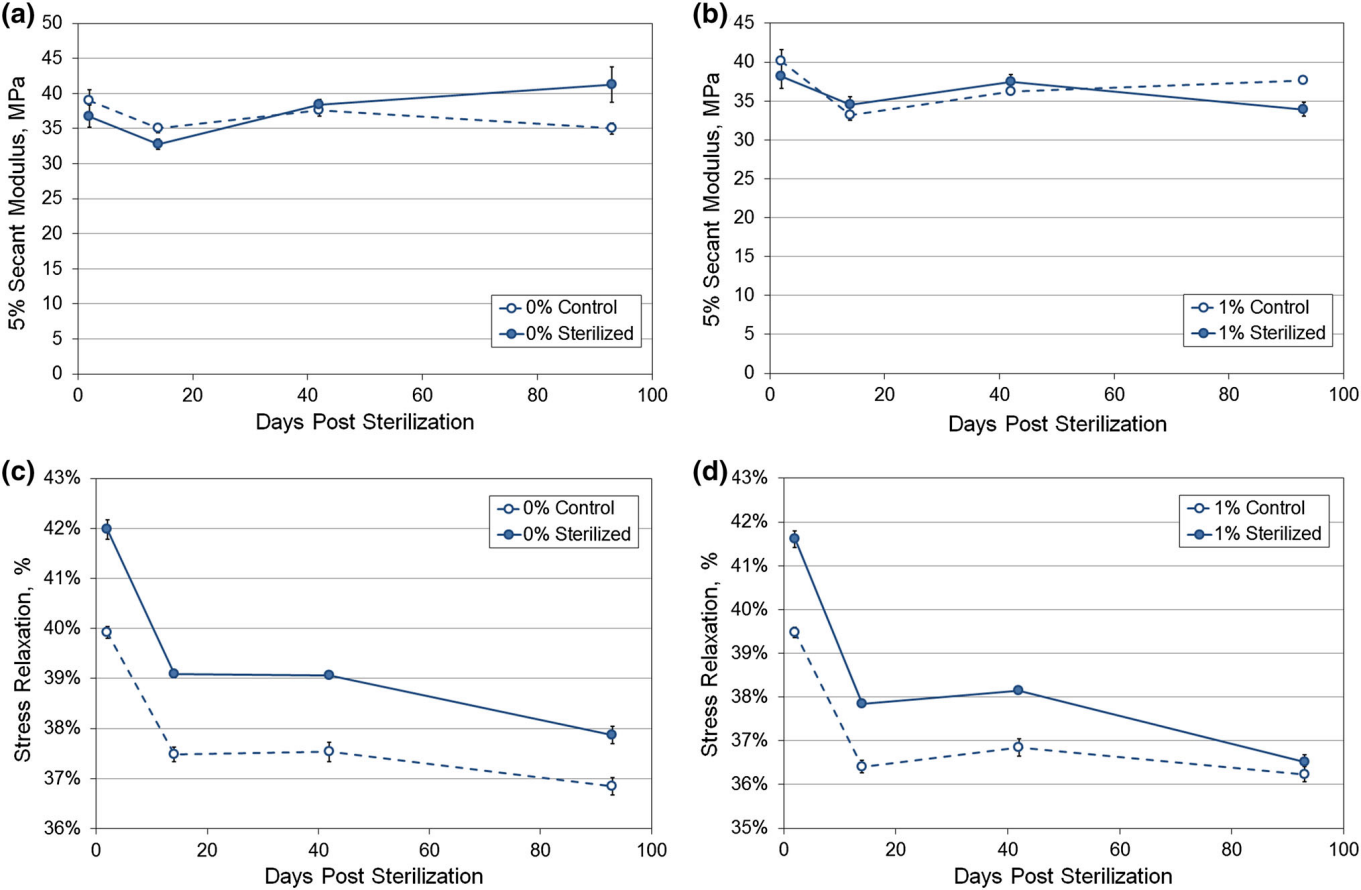
Results of the sterilization tests—secant modulus at 5% strain for the (a) neat, 0% and (b) composite 1.0% films and percentage stress relaxation for the (c) neat, 0% and (d) composite 1.0% films several days following gamma sterilization. Error bars represent ± SE.

In the sterilization study, the secant modulus at 5% strain for the 1.0% composite was 40.1 MPa compared to 39.0 MPa for the neat polymer (Figs. 8a and 8b). Two days following radiation exposure, there was a drop in modulus in both the neat and composite samples. There was an overall decrease in modulus in all polymers following 14 days. There was no significant difference (*p* > 0.05) in the secant modulus of either the neat or composite polymer at 93 days following sterilization.

Stress relaxation was highest in all samples 2 days following sterilization (Figs. 8c and 8d). The percentage stress relaxation following 30 s of hold at 45% strain was seen to increase in all samples following sterilization. Following 6 weeks after sterilization, the stress relaxation for the composite was similar to the control whereas the neat polymer still showed an increase compared to the control. Both the drop in stress relaxation with time and its increase following sterilization were significant (*p* < 0.05) following a two-way ANOVA test for both the neat and composite samples.

## Discussion

In this study, the suitability of carbon nanotube reinforced polyurethane composites was investigated as a potential leaflet material for transcatheter aortic valves. The tensile mechanical properties of the composites were determined in relation to the effects of nanotube dispersion time, long term (>30 days) immersion in water, strain rate and sterilization. Although higher aspect ratio nanotubes are expected to have greater reinforcement potential, they had a higher tendency to agglomerate and the Raman spectroscopy analysis confirmed higher structural defects through a higher ID/IG ratio. Consequently, composite development and testing was carried out with low aspect ratio multi-walled nanotubes only.

It was hypothesized that increased nanotube concentration in solution would correlate to an increase in polymer stiffness. However ultra-low concentrations of 0.125 and 0.25% w/w had a detrimental effect on the polymers mechanical properties at 5% strain. Although their thickness was slightly lower, it was not considered to impact their fatigue life, which was considerably higher than composites at higher concentrations. It is likely that the incorporation of a low amount of nanofillers has a negligible reinforcement effect but instead caused them to behave as defects.

A significant increase in the secant modulus of the composites was noted when the concentration increased to 0.5% w/w although there was no significant difference between the 0.5 and 1.0% composites. Gkikas *et al.* who investigated the effects of dispersion on the thermo-mechanical and toughness properties of nanotube composites also noted a similar outcome.^11^ Moreover, in this study, the neat polymer batch produced for strain rate testing had better mechanical properties than the composites, implying that there can be large variation in batches. These variations could be avoided in the future by improving the nanotube–polymer interface to ensure an even load transfer between the filler and matrix.

Most studies with these composites use very small filler quantities (<1.0% w/w) as their ultra-low densities and high aspect ratios give them a high volume to surface ratio in very small amounts. An increase in filler concentration leads to increased brittleness which can compromise the fatigue life of the material. Concentrations are also kept low since high van der Waal interaction between tubes facilitate agglomeration. Nanotube bundles reduce the surface area to volume ratio and introduce defects which would compromise rather than improve mechanical properties. A compromise needs to be achieved between reducing agglomerates without damaging the tubes and compromising reinforcement efficiency.

A popular technique to reducing agglomeration is through an ultrasonic bath or probe which generate shockwaves through the collapse of microbubbles. However, the high energy and heat used with ultrasound can cut nanotubes into shorter segments and introduce structural defects; a problem which only increases with prolonged sonication. In one study, no tube structure was observed following 5 h of sonication of single-walled nanotubes.^20^ The sonication frequency, use of a sonication probe, composite rheological properties and sample size could all affect the efficiency of ultrasonic dispersion.^11^ In addition, when developing polymeric heart valves through a dip-coating technique for example, the viscosity of the solution is critical in controlling the thickness of polymeric leaflets. Thus this study set out to define the ideal sonication time for a fairly viscous solution in a large bottle suitable for dip-coating. It was observed that increased dispersion time led to a progressive decrease in secant modulus. Consequently, the optimal dispersion time of the solution was defined to be 1 h.

Since 2002, the number of TAVI procedures in patients suffering from severe aortic stenosis has increased rapidly, enabling the elderly and frail to receive valve replacement treatment rather than medical therapy. Despite many TAVI routes now feasible which include trans-aortic and trans-apical delivery, trans-femoral TAVI remains the preference. However, major vascular bleeding is reported as one of the main complications from the PARTNER II trial, concurrent with a risk of stroke from dislodging plaque in calcific arteries. The situation is further complicated in women, children and smokers who generally have smaller arteries and in patients with tortuous femoral arteries.^27^ Consequently, it is critical to reduce the profile delivery of these valves to mitigate the risk from vascular complications.

TAVI valves are most commonly bioprosthetic valves, and pericardial leaflet thicknesses range from 200 to 400 *μ*m which is at least 4 times the thickness of the composites developed in this study. Bioprosthetic valves are also prone to early failure from calcification following necessary crosslinking treatment with glutaraldehyde to reduce immunogenicity and increase the mechanical strength. One TAVI technology has utilized a ‘dry membrane’ approach with porcine pericardium to reduce the thickness and packaging space but the long term effects of dry packaging tissue are yet to be determined.^24^ Polymeric valves can combine the durability of mechanical valves with the haemocompatibility of bioprosthetic ones.^33^ These valves, formed from polymers including polyurethane and silicone were previously limited to short term implantation use such as in left-ventricular assist devices due to complications from hydrolysis and oxidization. However, over the years there has been significant improvement in polymeric materials; polycarbonate urethane for example has greater resistance to hydrolysis than polyester urethane.^31^ The good mechanical strength of polyether urethane have also been combined with the biostability of silicone^22^, ^28^ to produce a potential polymeric composite material for heart valves (Elast-Eon™, AorTech, Surrey, United Kingdom).

In an earlier study (unpublished), glutaraldehyde treated porcine pericardium was measured to have thicknesses ranging from 170 to 292.5 *μ*m and an elastic modulus of 140.5 and 57.1 MPa in the longitudinal and radial directions of the fibers respectively. Although tissues like pericardium and polymers are both viscoelastic materials, their stress–strain behavior is very different. Pericardium is anisotropic and exhibits a toe region followed by a linear region in stress–strain graphs reminiscent of the crimped fiber structures unfolding. Conversely, polyurethane has a steep slope at low strains, which decreases with increasing strain. The loading behavior of pericardium and heart valve tissues enable a high degree of stretching at minimal pressure changes, which is beneficial for rapid opening and closure of the leaflets during the cardiac cycle. However, thickness is also a determining factor in valve performance, with thicker leaflets likely to impact transvalvular pressure gradients and leaflet closing efficiency.^1^ Consequently, reducing leaflet thickness can enhance hydrodynamic performance in polymeric valves.

Viscoelastic materials generally have higher stiffness’s at increased strain rates, caused by the polymer chains facing an insufficient time to rearrange and slide over each other. It was hypothesized then that there would be an increase in stiffness with increasing strain rate. This is a useful property to analyse with regards to heart valves which exhibit high strain rates in the duration of a cardiac cycle.^7^ In this study, the reinforcement effects of the carbon nanotubes in the 0.5 and 1.0% composites were more evident as the strain rate increased to the maximum rate tested.

Although medical grade polyurethanes are designed to be stable at standard sterilization procedures, the dosage level and sterilization technique adopted can impact the physical and mechanical properties. In one study, nanotubes embedded in polymer were found to reduce the loss in elastic modulus compared to the neat polymer as the nanotubes scavenged radicals mobilized in the radiation process.^25^ In this study, no significant change was observed in the secant modulus of composites following sterilization although a higher increase in modulus was noted with the neat polymer after 93 days. The stress relaxation of the composite returned to pre-sterilization values at the end of the study but not in the neat polymer. It is probable that the nanotubes dampened the effect of radiation on the composite.

This study has highlighted the potential of using nanotubes to reinforce ultra-thin leaflets for TAVI use, including the potential to dampen the effects from sterilization and reduce the aging effects of long term immersion in fluid. However, nanotubes could also compromise the fatigue life of the composites, which could have been caused by a poor fiber–matrix interface.^21^ Further investigations are needed to improve the nanotube–polymer composite interface to cope with the rapid, dynamic flexion of the leaflets during the cardiac cycle. Some studies have shown the addition of small quantities of carbon nanotubes to improve cyclic fatigue of polymers.^12^, ^19^ This highlights the possibility of improving fatigue resistance and elasticity of composite leaflets but the filler-matrix interface needs to be enhanced.

**Figure 9:**
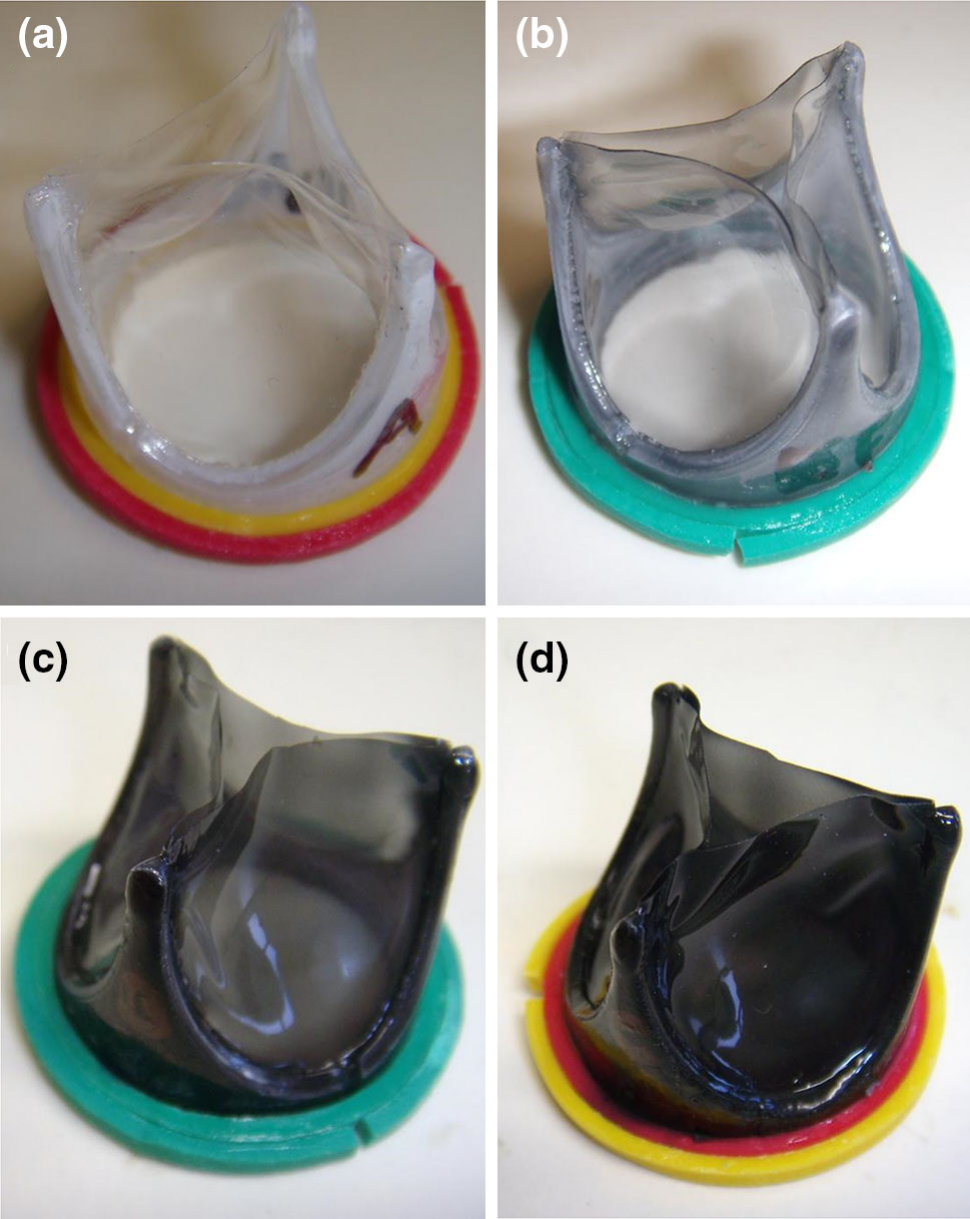
Dip coated, reinforced polymeric heart valves with (a) 0%, (b) 0.125%, (c) 0.5%, and (d) 1.0% nanotube concentrations.

Early attempts at nanotube functionalization through chemical and oxidization treatments were not found to improve the mechanical properties and may instead have caused fiber damage. Future attempts will be investigated through non-destructive means to improve fatigue life and one promising method is through dispersion using block copolymers.^34^ Presently, composite dip-coated heart valves with varying nanotube concentrations (Fig. 9) have been developed and are undergoing durability testing in a high-cycle fatigue tester to determine robustness. The hemodynamic properties of carbon nanotube polyurethane composites are also currently being investigated.

## Limitations

Composites were tested at room temperature to control testing between samples without any thermodynamic effects. However, as temperature influences the viscoelastic properties of the polymer, it will be necessary to investigate the thermodynamic properties of the composites at body temperature.

Another limitation is that this study did not investigate the functionalization of the nanotubes which could potentially increase its reinforcement potential, particularly at lower nanotube concentrations, without compromising the fatigue properties. Taking this study forward, the effect of nanotube functionalization through grafting of polymers or block copolymers will be investigated.

## Conclusion

In this study, the suitability of carbon nanotubes as reinforcement fillers to reduce the profile delivery of percutaneous heart valves, was investigated. The following conclusions were drawn:

1. Increased concentration of carbon nanotubes leads to a decreased fatigue life which would compromise the duration of the heart valves. However, composites with very low concentrations of 0.125 and 0.25% had worse mechanical properties at 5% strain than the neat polymer matrix. A balance needs to be achieved to optimize reinforcement potential whilst minimising impact on fatigue life.
2. The elastic modulus of composites increased more rapidly at increasing strain rates than the neat polymer. As heart valves exhibit very large strain rates, this property could be very useful in the development of polymeric composite leaflets.
3. There are many variables that can influence the reinforcement potential of carbon nanotubes in polymers. These include the sonication duration, which was addressed in this study but also nanotube purity, functionalization, nanotube–matrix interface, solution viscosity and temperature.

This study has identified the need for increased understanding of the nanotube–matrix interface. The impact of functionalization on reinforcement of these composites will be next investigated.
